# Surveillance and return to work of healthcare workers following SARS-CoV-2 Omicron variant infection, Sheffield, England, 17 January to 7 February 2022

**DOI:** 10.2807/1560-7917.ES.2022.27.11.2200164

**Published:** 2022-03-17

**Authors:** Mohammed Raza, Prosenjit Giri, Subhashis Basu

**Affiliations:** 1 Department of Virology, Sheffield Teaching Hospitals NHS Foundation Trust, Northern General Hospital, Sheffield, England; 2 Occupational Health Department, Sheffield Teaching Hospitals NHS Foundation Trust, Northern General Hospital, Sheffield, England

**Keywords:** Omicron, Covid, surveillance, healthcare, testing, work

## Abstract

The SARS-CoV-2 Omicron variant has challenged demands to minimise workplace transmission in healthcare settings while maintaining adequate staffing. Policymakers have shortened COVID-19 isolation periods, although little real-world data have evaluated the utility. Our findings from surveillance of 240 healthcare workers from Sheffield Teaching Hospitals, England, show that 55% of affected staff could return before day 10 of isolation with over 25% eligible on day 6, pending two successive negative antigen tests. This outcome is favourable for continuity of healthcare services.

Given the high transmissibility of the severe acute respiratory syndrome coronavirus 2 (SARS-CoV-2) Omicron (Phylogenetic Assignment of Named Global Outbreak (Pango) lineage designation B.1.1.529) variant, there remains concern particularly in the hospital environment as how to best balance the health and safety of inpatients while maintaining adequate staffing of healthcare workers (HCWs). To address HCW absences, policy changes in England [1] and the United States [2] have included shortened isolation periods following receipt of a positive test, with or without the use of daily testing to inform an appropriate exit. However, there are few published data to evaluate the appropriateness and outcomes of such policy changes. We present data from the Sheffield hospital network on daily testing and return to work of HCWs (after a positive SARS-CoV-2 test) over a 3-week period from when the minimum duration of isolation for HCWs in England was reduced from 10 to 5 full days, contingent upon two consecutive negative tests on days 5 and 6 [3].

## SARS-CoV-2 testing of healthcare workers

Sheffield Teaching Hospitals NHS Foundation Trust (STH) employs just over 17,000 personnel in various hospital and community-based settings and is one of the country’s largest hospital networks. Since May 2020, an on-site SARS-CoV-2 PCR testing hub has been available to all staff 7 days per week. It offers a drive-through service with self-swabbing test kits (nasal or combined nose/throat) and results for patient-facing HCWs are prioritised for rapid turnaround, i.e. usually well within 12 h [4]. Criteria for PCR testing include symptoms consistent with coronavirus disease (COVID-19), e.g. fever, myalgia, headache, runny nose, sore throat and/or persistent cough; this list has been periodically updated in line with emerging evidence of the symptom profile of new variants of concern. Staff who were asymptomatic could also utilise the testing hub if they were contacted by the United Kingdom Health Security Agency as a suspected household or community contact of a confirmed COVID-19 case.

## Adaptation of isolation requirements following changes in government policy

During much of the COVID-19 pandemic, the isolation requirements in England for HCWs with a positive SARS-CoV-2 test have been a 10-day period, which allowed a return to work on day 11 post-infection. Given the strained healthcare continuity and apparent reduction in severity of the Omicron variant, a change in English Government policy was enacted on 17 January 2022 that reduced the original 10-day isolation to 5 full days [1]. This new policy enabled a return on day 6, at the earliest, provided the individual had two successive negative SARS-CoV-2 rapid antigen tests.

**Figure 1:**
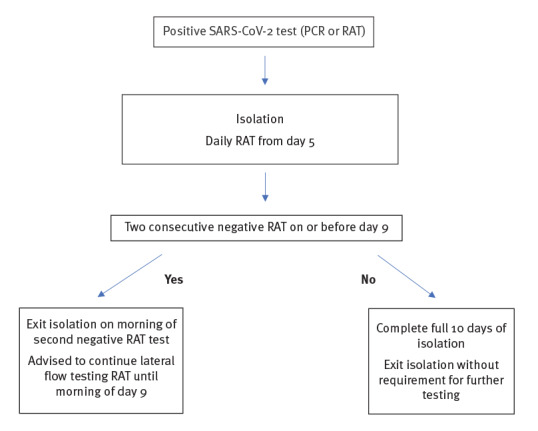
Flowchart for the SARS-CoV-2 testing process for healthcare workers, Sheffield Teaching Hospitals, Sheffield, England, from 17 January 2022

RAT: rapid antigen test; SARS-CoV-2: severe acute respiratory syndrome coronavirus 2.

At STH, those individuals who still tested positive at day 9 were instructed to complete a full tenth day of isolation before returning to work without the requirement for further testing (Figure 1), given that HCWs could be positive over a longer period but are unlikely to be infectious [5]. Asymptomatic HCWs testing positive through the hub following household and community contact tracing were also required to follow the process outlined in Figure 1.

After a government policy change enacted on 17 January 2022, healthcare workers could return to work after 5 days of isolation pending two negative RATs. Prior to this, the isolation period was 10 days, with the earliest return-to-work day on day 11.

## Outcome after adaptation to policy change

The Virology and Occupational Health departments of STH record positive SARS-CoV-2 test results and of consecutive negative results before returning to work. We analysed these available data between 17 January and 7 February 2022. During the 3-week period, 312 STH HCWs returned positive SARS-CoV-2 PCR tests. Of these, self-reported rapid antigen test data during isolation from 72 individuals were incomplete and we were unable to determine the point at which two successive negative results were attained or the result of their day 9 test. Accordingly, data from 240 individuals were included in this analysis.

**Figure 2:**
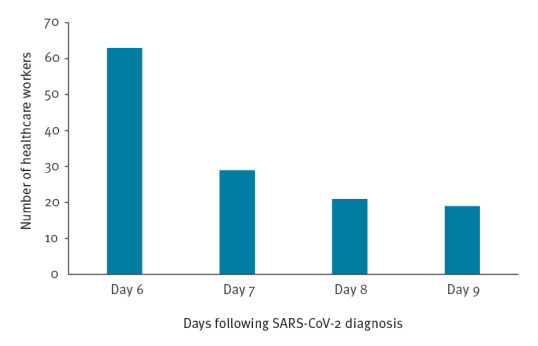
Number of healthcare workers eligible to return to work on consecutive days following a positive SARS-CoV-2 test, Sheffield Teaching Hospitals, Sheffield, England, 17 January–7 February 2022 (n = 132)

The mean age of individuals in our sample was 42 years (range: 21–70) with 202 (84%) female HCWs. One hundred and eight individuals (45%) continued to record positive rapid antigen tests up to and including the morning of day 9 post-diagnosis. For the 132 HCWs that returned before day 10, the median was day 6, on which 63 HCWs were eligible to return. A further 29, 21 and 19 were able to return on days 7, 8 and 9, respectively (Figure 2). No differences were observed for median return date by sex or age, when stratified into those aged 40 years and above vs younger HCWs (data not shown).

SARS-CoV-2: severe acute respiratory syndrome coronavirus 2.

Of the total 240 healthcare workers studied, 132 had two consecutive negative SARS-CoV-2 rapid antigen tests and could return to work on day 9 or before (day 6, n = 63; day 7, n = 29; day 8, n = 21; day 9, n = 19).

Two hundred (83%) HCWs reported an asymptomatic SARS-CoV-2 infection. Of the 40 HCWs declaring COVID-19 symptoms, most described upper respiratory symptoms such as sore throat and coryza. Nine described systemic symptoms such as fever, myalgia, and breathlessness. Of the 240 HCWs, 224 (93%) reported having received at least two doses of vaccine at least 2 weeks before they recorded a positive SARS-CoV-2 test.

## Implications of policy change

During our 3-week observation, the policy change allowed 55% (n = 132) of affected HCWs to return to work earlier; notably, over 25% (n = 63) of HCWs could return by day 6. This equated to 532 days of additional healthcare provision. Incidentally, 23 individuals who tested themselves of their own accord with SARS-CoV-2 antigen tests on a daily basis following their positive result returned successive negative tests before day 6, with the earliest second negative result on day 3. Under the policy however, these individuals could not exit their isolation until completing 5 full days This change in policy was therefore beneficial for staffing continuity and was not associated with a spike in SARS-CoV-2 positive cases among hospital inpatients during the same period, i.e. 293 and 209 SARS-positive inpatient cases on 17 January and 7 February 2022, respectively. Although 45% of HCW (108/240) continued to test positive by rapid antigen test on day 9, evidence suggests that the likelihood of infectiousness beyond day 10 is very low [5]. Accordingly, these HCW returned on day 11 following the first positive test.

## Ethical statement

Ethical approval was not necessary because all surveillance data used are mandatorily collected according to the law.

## Discussion

With the emergence of the SARS-CoV-2 Omicron variant, critical services such as health and social care continue to employ daily SARS-CoV-2 testing regimens as a means of surveillance and reduction of virus transmission by identifying and isolating infectious individuals at an early stage. Throughout the COVID-19 pandemic, hospitals in countries without widespread access to rapid testing and robust test-out-of-isolation surveillance systems to reduce the risk of within-facility transmission have faced difficulties in maintaining staffing [1,2]. These challenges are likely more substantial with Omicron, with affected countries consistently reporting rapid community transmission of Omicron with reproduction numbers far higher than previous variants, in part because of immuno-evasive properties [6-8]. It is plausible that these difficulties are also greater in countries with low vaccine coverage and population immunity.

Implementation of a policy to reduced isolation time for HCWs following a positive SARS-CoV-2 PCR test allowed a more rapid return to work and improved continuity for our hospital network. During a period of high transmission, this change in policy saved 532 days of HCW absence in only 3 weeks’ time. We found that most HCW examined during the study period were asymptomatic following a positive SARS-COV-2 test. Since data regarding infectiousness of individuals with asymptomatic infections, particularly for the Omicron variant, are limited, further reinforcement is needed for testing as an adjunct for exit from isolation [9, 10]. Policies which shorten isolation periods following a positive SARS-CoV-2 test may not be appropriate for HCW caring for clinically vulnerable populations at risk for severe disease. Accordingly, our hospital network, in line with other NHS Trusts, has specific policies for these areas where HCWs are required to demonstrate viral clearance beyond day 11.

The percentage of individuals who received at least two doses of vaccine in our sample were reflective of the broader rates of vaccine uptake within the hospital, of over 95%. In line with English government policy, HCWs under 40 years of age would have received two initial doses of mRNA vaccine, whereas those over 40 years would have received a viral vector-based vaccine. All booster doses are mRNA-based unless contraindicated. We anticipate our findings are at least generalisable to other healthcare environments with similarly high vaccination coverage. However, the degree of waning of vaccine-derived immunity, even after additional ‘booster’ doses on transmission and illness severity is uncertain but evidence so far suggests this occurs [11]. Therefore, it is plausible that policies, such as the one presented here, may need to be in place for some time to reduce disruption in staffing. Further work should expand on these areas and ascertain any differences in return-to-work times between symptomatic and asymptomatic individuals, as well as by vaccination status.

Limitations of this analysis include the small sample size and short 3-week coverage period. Nonetheless, our findings provide a useful first step in broadly assessing the effectiveness and safety implications of current COVID-19 isolation policies designed to balance the risks of returning potentially infected HCW to work with those of patient safety from chronic understaffing. In addition, only a proportion of tests from our laboratory are routinely sampled for genomic analysis, and thus we cannot be certain that all of our positive PCR test results were the Omicron variant. Nonetheless, genomic surveillance data from the time in Sheffield, England indicated that Omicron lineages BA.1 and BA 1.1 accounted for 98.8% of positive cases at the time [12]. Therefore, we anticipate our results are broadly generalisable to other Omicron-dominant settings and can help inform approaches in balancing the competing demands of staffing levels with patient and colleague safety.

## Conclusions

Although our findings provide some reassurance regarding the safety of a change in policy to shorten isolation periods to less than 10 full days, we advise caution in applying return-to-work policies that include shortened isolation periods, particularly without the use of testing. We also recommend careful consideration of the safety of such policies in community and healthcare settings with clinically vulnerable individuals who are older or immunocompromised, or where there is low vaccine coverage among HCWs and/or patients. The change in isolation policy and testing process enabled over half of our HCWs who tested positive for SARS-CoV-2 during this 3-week period to return earlier than previously, easing our staffing pressures whilst providing medical reassurance regarding the safety of patients and work colleagues.
